# Serum Angiopoietin 2 acts as a diagnostic and prognostic biomarker in hepatocellular carcinoma

**DOI:** 10.7150/jca.56436

**Published:** 2021-03-05

**Authors:** Junjie Ao, Tetsuhiro Chiba, Hiroaki Kanzaki, Kengo Kanayama, Shuhei Shibata, Akane Kurosugi, Terunao Iwanaga, Motoyasu Kan, Takafumi Sakuma, Na Qiang, Yaojia Ma, Ryuta Kojima, Yuko Kusakabe, Masato Nakamura, Kazufumi Kobayashi, Soichiro Kiyono, Naoya Kanogawa, Tomoko Saito, Ryo Nakagawa, Takayuki Kondo, Sadahisa Ogasawara, Eiichiro Suzuki, Shingo Nakamoto, Ryosuke Muroyama, Akinobu Tawada, Jun Kato, Tatsuo Kanda, Hitoshi Maruyama, Naoya Kato

**Affiliations:** 1 Department of Gastroenterology, Graduate School of Medicine, Chiba University, 1-8-1 Inohana, Chuo-ku, Chiba 260-8670, Japan.; 2 Department of Gastroenterology and Hepatology, Nihon University School of Medicine, 30-1 Oyaguchi-Kamicho, Itabashi-ku, Tokyo 173-8610, Japan.; 3 Department of Gastroenterology, Juntendo University School of Medicine, 2-1-1 Hongo, Bunkyo-ku, Tokyo 113-8421, Japan.

**Keywords:** Hepatocellular carcinoma, biomarker, angiopoietin 2, enzyme-linked immunosorbent assay

## Abstract

Hepatocellular carcinoma (HCC) is typically accompanied by abundant arterial blood flow. Although angiogenic growth factors such as Angiopoietin 2 (Ang2) play a central role in tumor angiogenesis in HCC, the role of serum Ang2 as a biomarker in HCC remains unclear. In this study, we aimed to investigate the potential of Ang2 as a diagnostic and prognostic biomarker in HCC using a sandwich enzyme-linked immunosorbent assay (ELISA). The median Ang2 levels in controls (n=20), chronic liver disease patients (n=98), and HCC patients (n=275) were 1.58, 2.33, and 3.53 ng/mL, respectively. The optimal cut-off value of Ang2 was determined as 3.5 ng/mL by receiver operating curve analysis. The sensitivity, specificity, and accuracy of Ang2 for HCC detection were 50.9, 83.7, and 59.5%, respectively. Spearman's rank correlation coefficient analysis demonstrated only a weak correlation between Ang2 serum levels and alpha-fetoprotein (AFP) or des-gamma-carboxy prothrombin (DCP) serum levels. The diagnostic value of Ang2 was comparable to those of other existing markers. In addition, 24 out of 73 patients with normal AFP and DCP levels (32.9%) demonstrated abnormally high Ang2 levels (≥3.5 ng/mL). Although no significant difference in overall survival was found between Ang2^high^ and Ang2^low^ patients with curative ablation therapy, recurrence-free survival (RFS) in Ang2^high^ patients was observed to be significantly shorter than those in Ang2^low^ patients. Multivariate analysis demonstrated that high serum Ang2 levels (≥3.5 ng/mL) and the presence of multiple tumors were poor prognostic factors. In conclusion, our findings indicate that serum Ang2 is a potential novel biomarker for both diagnosis and prognosis in HCC.

## Introduction

Tumor angiogenesis is one of the representative hallmarks of cancer that contributes to tumor enlargement and metastatic dissemination 1. While a small mass of solid tumors ≤ 1-2 mm in diameter in what is called tumor dormancy does not necessarily require vessels 2, neovascular vessel formation is indispensable for the expansion of dormant micro-tumors beyond 1-2 mm in diameter. Angiogenesis in tumors is regulated by the balance between pro- and anti-angiogenic factors. Angiopoietin/Tyrosine kinase with Ig and EGF homology domains 2 (TIE2) signaling, vascular endothelial growth factor (VEGF)/VEGF receptor (VEGFR) signaling, and platelet-derived growth factor (PDGF)/PDGF receptor (PDGFR) signaling serve as angiogenic signaling 3-5. In contrast, endogenous angiogenic inhibitors, such as angiostatin, endostatin, and thrombospondin-1 function as suppressors of angiogenesis 6. Angiogenic switch is caused by the imbalance between pro- and anti-angiogenic factors skewing towards a pro-angiogenic outcome, which results in transition from tumor dormancy to tumor progression 7, 8.

The angiopoietin (Ang) family, consisting of Ang1, Ang2, and Ang3/4, are comprised of ligands of the tyrosine kinase with Ig and EGF homology domain (Tie) 1 and Tie2 9. Ang1 is mainly produced by vascular smooth-muscle cells and functions as an agonist for Tie2 via its autophosphorylation 10. In contrast, Ang2 is predominantly secreted from tumor cells and acts as an antagonist for Tie2. The Ang2-Tie2 pathway suppresses interactions between endothelial cells and mural cells (pericytes and vascular smooth muscle cells) thereby promoting vascular remodeling 11. Interestingly, Ang2 induces endothelial cell death and vessel regression in the absence of VEGF, whereas it promotes the remodeling of the vessels and vascular sprouting of new blood vessels in the presence of VEGF 12, 13. Ang2 was reported to be highly expressed in hypervascular HCC compared to hypovascular HCC 14. It has been also demonstrated that loss-of-function of Tie2 resulted in a decrease in tumorigenicity with neovascularization in mouse tumor models 15. These findings indicate that Ang2-Tie2 pathway plays a crucial role in both the neovascularization and progression of HCC.

In this study, we examined the serum Ang2 levels of 275 consecutive HCC patients as well as controls and chronic liver disease (CLD) patients using a sandwich enzyme-linked immunosorbent assay (ELISA). Subsequently, we compared the diagnostic ability of Ang2 with that of the pre-existing tumor markers. Moreover, the utility of Ang2 as a prognostic indicator was also investigated in HCC patients treated with curative ablation therapy.

## Materials and Methods

### Collection and analyses of blood samples

Blood samples were collected from 275 patients who were primary cases without any treatments for HCC at Chiba University hospital between 2014 and 2017. The sera of 98 CLD patients without HCC and those of 20 non-CLD patients (controls) were also collected. After obtaining informed written consent, we analyzed the stored blood samples. Laboratory data and imaging findings were also acquired from their medical records. This study was approved by the Research Ethics Committees of the Graduate School of Medicine, Chiba University (approval number: 3024).

### Diagnoses of chronic hepatitis, cirrhosis, and HCC

The diagnosis of CLD, including chronic hepatitis and cirrhosis, was based on the laboratory data, clinical manifestations, and/or histological findings 16. HCC was diagnosed on the basis of contrast-enhanced imaging findings and/or histological analysis of tumors according to the diagnostic criteria of the American Association for the Study of Liver Diseases 17.

### Determination of serum Ang2, AFP, and DCP concentrations

Serum Ang2 levels of the controls, CLD patients, and HCC patients were measured by a sandwich ELISA according to the manufacturer's instructions (R&D Systems, Inc., MN, USA). Serum alpha-fetoprotein (AFP) and des-g-carboxy prothrombin (DCP) levels were determined by Lumipulse^®^ L2400 (Fujrebio Inc., Tokyo, Japan) using chemiluminescence enzyme immunoassay (CLEIA). Serum Ang2 levels of HCC patients were determined using sera collected during the 1-month period before therapeutic intervention. The serum Ang2 levels of 45 patients treated with complete ablation were also analyzed at some point in the recurrence-free period based on radiological findings.

### Statistical analyses

All statistical analyses were performed using SPSS statistical software (SPSS version 24). Data are expressed as the mean ± standard deviation (SD). Statistical differences between the two groups were analyzed by the χ^2^-test or Mann-Whitney *U* test. The correlation between each marker was determined using Spearman's rank correlation coefficient. The area under the curve (AUC) values were determined with receiver-operating characteristics (ROC) analysis. Overall survival (OS) and recurrence-free survival (RFS) were calculated using the Kaplan-Meier method and compared using the log-rank test. The prognostic relevance of clinical variables was evaluated by uni- and multivariate cox regression analysis. *P*‐values <0.05 were considered significant.

## Results

### Patients' characteristics

A total of 275 primary HCC patients were comprised of 198 males (72.0%) and 77 females (28.0%), with a median age of 72 years (range: 40-92 years) (Table 1). Chronic liver damage was due to HBV (n=31), HCV (n=138), and others (n=106). The patients were classified as either class A (n=222), class B (n=44), and class C (n=9) according to the Child-Pugh classification. The number of patients with Union for International Cancer Control (UICC) stages I, II, III, and IV were 128 (46.5%), 68 (24.7%), 59 (21.5%), and 20 (7.3%), respectively.

The CLD patients consisted of 62 males (63.3%) and 36 females (36.7%) whose median age was 67 years (range: 35-84 years). Among them, 32 patients (32.7%) were diagnosed with cirrhosis. Chronic liver damage was due to HBV (n=21), HCV (n=48), and others (n=29). The patients were classified as either class A (n=86), class B (n=11), and class C (n=1) based on the Child-Pugh classification. The controls consisted of 14 males and 6 females whose median age was 60 years (range: 28-84 years).

### Serum Ang2 levels in control, CLD, and HCC patients

**Figure 1:**
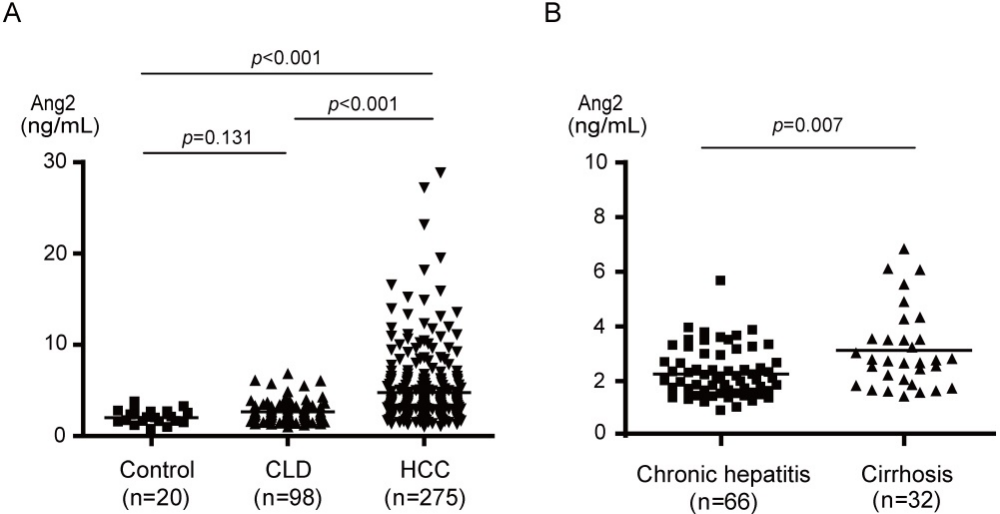
** Serum Ang2 levels in controls, CLD patients, and HCC patients.** (A) Serum Ang2 levels in HCC patients were significantly higher than those in controls (p<0.001) and CLD patients (p<0.001). (B) Serum Ang2 levels in cirrhotic patients were significantly higher than those in patients with chronic hepatitis (p=0.007).

The results of ELISA demonstrated that the median serum Ang2 levels in control, CLD, and HCC patients were 1.99, 2.26, and 3.83 ng/mL, respectively (Fig. 1A). The Ang2 level in HCC patients, ranging from 1.34 to 28.81 ng/mL, was significantly higher than those in both controls (*p*<0.001) and CLD patients (*p*<0.001). There was no significant difference between controls and CLD patients. However, serum Ang2 levels in cirrhotic patients were significantly higher than those in chronic hepatitis patients (*p*=0.007) (Fig. 1B).

### ROC analysis

**Figure 2:**
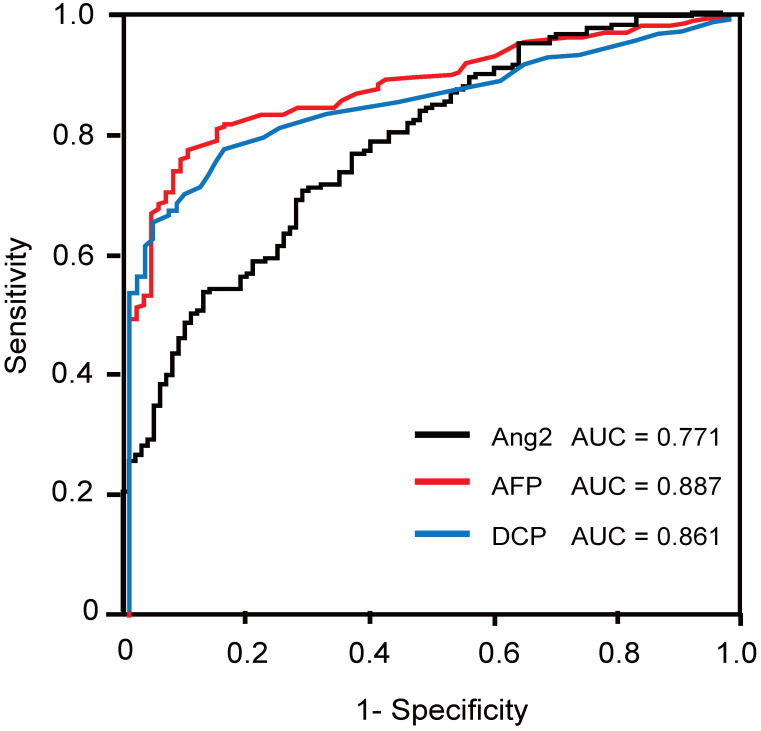
ROC curves of Ang2, AFP, and DCP for the detection of HCC.

Next, we conducted ROC analysis of Ang2 and existing serum markers, namely AFP and DCP, and compared their diagnostic ability (Fig. 2). The AUC values of Ang2, AFP, and DCP for HCC detection were 0.771, 0.887, and 0.861, respectively (Fig. 2). The optimal cut-off value was determined as 3.5 ng/mL using the Youden index. Likewise, those of AFP and DCP were decided as 20 ng/mL and 40 mAU/mL, respectively.

### Diagnostic value of Ang2 for HCC detection

**Figure 3:**
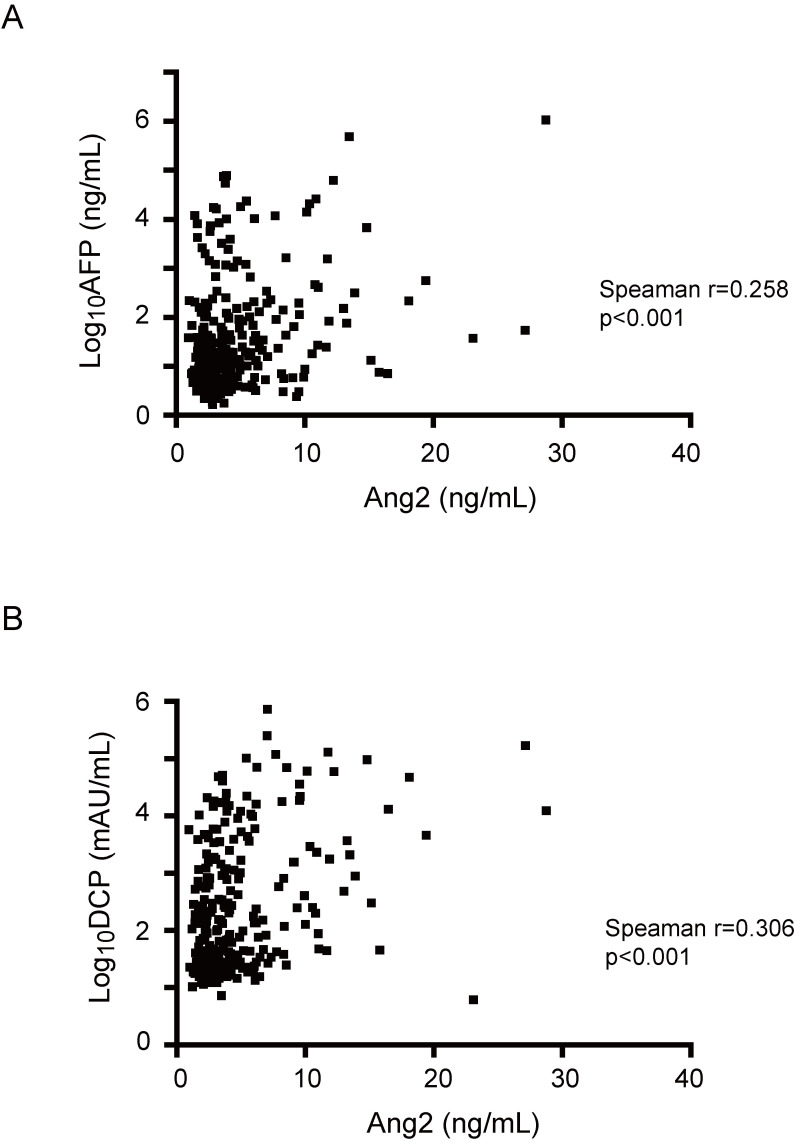
** Correlation between serum levels of Ang2 and AFP or DCP.** (A, B) Spearman's rank correlation analyses showed a weak positive linear correlation of serum Ang2 levels with AFP (A) or DCP (B).

To examine the correlation between Ang2 and existing markers such as AFP and DCP, Spearman's rank correlation coefficient analyses were performed (Fig. 3). The results demonstrated a weak correlation between Ang2 serum levels and AFP (Spearman r=0.258, *p*<0.001) or DCP (r=0.306, *p*<0.001) serum levels. These results indicate a weak negative correlation between Ang2 and AFP or DCP.

Next, we evaluated the diagnostic value of Ang2 with AFP and DCP. The sensitivity, specificity, and accuracy of Ang2 for HCC diagnosis were 50.9, 83.7, and 59.5%, respectively (Table 2). Although the sensitivities of AFP and DCP for HCC diagnosis were 43.6 and 61.8%, respectively, additional Ang2 measurement increased their sensitivity to 68.0 (*p*<0.001) and 76.0% (*p*<0.001), respectively. Similarly, the measurement of Ang2 added to AFP and DCP resulted in an increase in sensitivity from 73.5 to 82.2% (*p*=0.012). Of importance, 24 out of 73 HCC patients with normal AFP and DCP could be detected by additional Ang2 measurement. Together, Ang2 demonstrated not only diagnostic value but also a supplementary diagnostic effect in addition to existing markers.

### Association between serum Ang2 levels and clinicopathological features

We also investigated the association between serum Ang2 levels and clinicopathological features (Table 3). The patients were categorized as Ang2^high^ (≥3.5 ng/mL) or Ang2^low^ (<3.5 ng/mL) based on the serum Ang2 levels. Of importance, Ang2^high^ was significantly correlated with both cirrhosis (*p*=0.012) and Child-Pugh classification B or C (*p*<0.001). AFP and DCP levels were both significantly higher in Ang2^high^ patients than those in Ang2^low^ patients (*p*<0.001). Ang2^high^ was also significantly correlated with the presence of multiple tumors (*p*<0.001), a large tumor diameter (*p*=0.012), macrovascular invasion (*p*=0.008), and extrahepatic metastasis (*p*=0.028). Consistent with these findings, high serum Ang2 levels were also significantly correlated with UICC stage progression (*p*<0.001). Together, the elevation of serum Ang2 levels was correlated with not only liver damage but also tumor progression.

### Prognosis based on serum Ang2 levels

**Figure 4:**
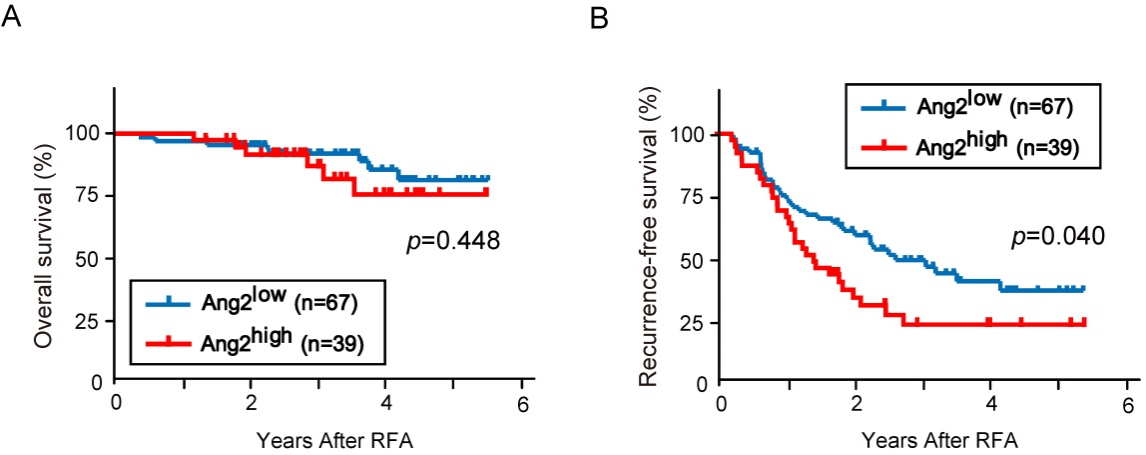
** Kaplan-Meier analyses based on serum Ang2 levels.** (A) There was no significant difference in OS between Ang2^low^ and Ang2^high^ patients (p=0.448). (B) RFS in Ang2^high^ patients was significantly shorter than that in Ang2^low^ patients (p=0.040).

Among the 275 patients analyzed in this study, 106 were treated with curative radiofrequency ablation (RFA) based on post-treatment imaging findings. There were no significant differences in clinical variables between Ang2^low^ (n=67) and Ang2^high^ (n=39) patients (Table 4). We then conducted Kaplan-Meier analysis of recurrence and survival according to the serum Ang2 levels. No significant difference in OS between Ang2^low^ and Ang2^high^ patients (*p*=0.448, Fig. 4A). However, RFS in Ang2^high^ patients was significantly shorter than that in Ang2^low^ patients (median RFS: 17.4 vs. 31.7 months, *p*=0.040, Fig. 4B).

Next, we examined the prognostic significance of clinical variables, including age, sex, HCV infection, Child-Pugh classification, cirrhosis, serum AFP, serum DCP, serum Ang2, tumor numbers, and the maximal tumor diameter (Table 5). The variables with *p*<0.10 on univariate analysis (cirrhosis, serum AFP, serum Ang2, and the number of tumors) were subjected to multivariate analysis. Multivariate Cox's regression analysis demonstrated that both serum Ang2 levels and tumor numbers showed a significant correlation with RFS. These results indicate that high serum Ang2 levels and the presence of multiple tumors were poor prognostic factors after curative treatment.

## Discussion

A biomarker is defined as a characteristic that is objectively measured and evaluated as an indicator of normal biologic processes, pathogenic processes, or pharmacologic responses to therapeutic intervention 18. Biomarkers are extremely important especially in the field of cancer care because they enable us to assess the carcinogenic risk, make an early diagnosis, predict the treatment response, and determine the prognosis. AFP and DCP have been widely utilized as serum biomarkers for HCC. However, the detection rate of HCC with AFP or DCP remains at only 50-60% when used alone, and approximately 70% when used simultaneously 19, 20. It is particularly difficult to not only diagnose patients but also monitor them after treatment when HCC is negative for both AFP and DCP 21, 22. Although some prospects have been reported, there is still a great need for biomarkers with good diagnostic performance 23, 24.

VEGF is the most representative angiogenic factor in HCC and its expression is induced by hypoxia and cell proliferation signals 25. Immunohistochemical (IHC) studies have shown that VEGF expression is not confined to the tumor area but also in surrounding non-tumor areas in a similar manner 26, 27. Concordant with these findings, there was no significant difference in the median serum VEGF levels between HCC patients and cirrhotic patients without HCC 28. In contrast, Ang2 has been observed to be more highly expressed in tumor tissues than in surrounding non-tumor tissues and to be less expressed in the normal liver 29. Therefore, we focused on the potential of Ang2 as a biomarker and examined the serum Ang2 levels of HCC patients using sandwich ELISA.

Serum Ang2 levels in HCC patients were significantly higher than those in controls (*p* < 0.001) and CLD patients (*p* < 0.001) as expected. While the specificity of Ang2 for HCC detection was slightly lower than that of AFP or DCP, its sensitivity and accuracy were superior to AFP and inferior to DCP. Recently, meta-analyses on the diagnostic performance of serum biomarkers such as Glypican-3 (GPC3) and Dickkopf-1 (DKK1) have been reported 30, 31. The sensitivity and specificity of GPC3 were 55% and 58%, respectively, and those of DKK1 were 65% and 94%, respectively. Similar to Ang2, the diagnostic ability of these markers is not necessarily better than that of AFP and DCP. Given that molecular tumor heterogeneity was demonstrated in HCC, it might be more practical to use a combination of markers rather than a single marker.

It has been well-known that AFP and DCP in HCC patients sometimes remain in the normal range 21, 22. Since only a weak correlation between Ang2 and AFP or Ang2 and DCP was observed, we examined whether adding Ang2 to AFP and DCP would increase the rate of diagnosing HCC. As a result, additional measurement of Ang2 was found to improve the sensitivity of either AFP or DCP individually and both AFP and DCP together. We then sought to unveil the difference in clinical features between Ang2^high^ patients with normal AFP/DCP (n=24) and Ang2^low^ patients with normal AFP/DCP (n=49). However, there were no significant differences in clinical variables between both groups (data not shown). Further analyses would be necessary in a larger number of patients.

Our ELISA data demonstrated that Ang2 levels in patients with cirrhosis were significantly higher than those in patients with chronic hepatitis. It has been reported that Ang2 mRNA was detected in the strands of fibrous tissue, but not in hepatocytes in cirrhotic patients 32. Hepatic stellate cell activation via the Ang2/Tie2 signaling axis might contribute to fibrosis 33. In the chronic treatment of a CCl4 -treated rat model, anti-Ang2 treatment was reported to successfully reduce liver fibrosis 34. Thus, the use of Ang2 as a biomarker and therapeutic target for hepatic fibrosis seems promising.

Next, we compared the clinicopathological features between Ang2^high^ and Ang2^low^ patients. Ang2^high^ was significantly correlated with large and multiple tumors, stage progression, and aggressive phenotypes characterized by macrovascular invasion and extrahepatic metastasis. We then analyzed the prognosis of early-stage patients receiving RFA. Our Kaplan-Meier analyses demonstrated that RFS, but not OS, in Ang2^high^ patients was significantly poorer than that in Ang2^low^ patients. Although serum Ang2 levels in patients treated with transarterial radioembolization and hepatic venous Ang2 levels were also associated with OS 35, 36, our study is the first to show that serum Ang2 levels are associated with prognosis after ablation therapy for HCC. It has been also reported that serum VEGF and Ang2 levels are associated with OS in patients treated with sorafenib 37. Considering that the effect of Ang2 on vascular endothelial cells is different in the presence and absence of VEGF 12, 13, simultaneous measurement of Ang2 and VEGF may be useful in predicting the prognosis of some patients with advanced HCC.

In conclusion, our study demonstrated that Ang2 is a potential novel biomarker for both diagnosis and prognosis in HCC. It has been demonstrated that baseline Ang2 level is related to the treatment effect of sorafenib and lenvatinib 38, 39. Considering the central role of angiogenesis-inhibiting therapy in recent drug therapies for HCC 40, 41, it may be of marked significance as a biomarker for predicting therapeutic efficacy and making a prognosis.

## Figures and Tables

**Table 1:** Baseline characteristics of the patients with HCC

Characteristics	Value (n=275)
Age (years)*	71 (40-92)
Sex (male/female)	198/77
Etiology HBV/HCV/others	31/138/106
Liver damage (CH/LC)	48/227
Child-Pugh Classification A/B/C	222/44/9
ALT (IU/L)*	34 (8-1,920)
ALB (g/dL)*	3.7 (2.1-4.9)
T-Bil (mg/dL)*	0.9 (0.3-9)
PLT (×10,000/μL)*	11.5 (3-76.5)
PT (%)*	93 (21-131)
FIB-4 index*	5.17 (0.74-84.71)
AFP (ng/mL)*	15.6 (1.6-1,032,500)
DCP (mAU/mL)*	86 (6-700,090)
Tumor numbers (solitary/multiple)	134/141
Maximal tumor diameter (≤20/>20 mm)	98/177
Macrovascular invasion (yes/no)	29/246
Extrahepatic metastasis (yes/no)	13/262
UICC stage (I/II/III/IV)	128/68/59/20

*Median (range).

**Table 2:** Sensitivity, specificity, and accuracy of serum Ang2 and existing markers in HCC cases

	Sensitivity (%)	Specificity (%)	Accuracy (%)
**Single markers**			
AFP	43.6	99.0	58.2
DCP	61.8	96.9	71.0
Ang2	50.9	83.7	59.5
**Double markers**			
AFP and DCP	73.5	96.0	79.4
AFP and Ang2	68.0	82.7	71.8
DCP and Ang2	76.0	80.6	77.2
**Triple markers**			
AFP, DCP, and Ang2	82.2	79.6	81.5

**Table 3:** Differences in clinical features between Ang2^low^ and Ang2^high^ patients

Characteristics	Ang2^low^ (<3.5 ng/mL, n=135)	Ang2^high^ (≥3.5 ng/mL, n=140)	*p-*value
Age (years)*	72 (40-88)	69.5 (43-92)	0.159
Sex (male/female)	99/36	99/41	0.727
Etiology HBV/HCV/others	15/74/46	16/64/60	0.288
Liver damage (CH/LC)	32/103	16/124	0.012
Child-Pugh classification (A/B/C)	127/8/0	95/36/9	<0.001
AFP (ng/mL)*	10.6 (1.6-16,925)	24.9 (1.7-1,032,500)	<0.001
DCP (mAU/mL)*	40 (10-47,053)	236 (6-700,090)	<0.001
Tumor numbers (solitary/multiple)	80/55	54/86	<0.001
Maximal tumor diameter (≤20/>20 mm)	55/80	36/104	0.012
Macrovascular invasion (yes/no)	7/128	22/118	0.008
Extrahepatic metastasis (yes/no)	2/133	11/129	0.027
UICC stage (I/II/III/IV)	79/35/16/5	49/33/43/15	<0.001

*Median (range).

**Table 4:** Differences in clinical features between Ang2^low^ and Ang2^high^ patients treated with curative ablation therapy

Characteristics	Ang2^low^ (<3.5 ng/mL, n=67)	Ang2^high^ (≥3.5 ng/mL, n=39)	*p-*value
Age (years)*	70 (40-88)	73 (50-87)	0.467
Sex (male/female)	49/18	21/18	0.070
Etiology HBV/HCV/others	6/50/11	4/24/11	0.317
Liver damage (CH/LC)	17/50	3/36	0.047
Child-Pugh classification (A/B/C)	63/4/0	33/5/1	0.190
Fib-4 index*	5.17 (1.19-19.91)	6.28 (2.39-15.69)	0.013
AFP (ng/mL)*	8.6 (1.8-1,377.5)	15.2 (2.6-220.4)	0.075
DCP (mAU/mL)*	28 (11-17,588)	36 (14-3,785)	0.138
Ang2 (ng/mL)*	2.33 (1.26-3.47)	4.83 (3.52-11.08)	<0.001
Tumor numbers (solitary/multiple)	52/15	28/11	0.662
Maximal tumor diameter (≤20/>20 mm)	46/21	24/15	0.594
UICC stage (I/II)	52/15	28/11	0.662

*Median (range).

**Table 5:** Uni- and multivariate analyses of factors predicting RFS

	Univariate analysis		Multivariate analysis	
	Hazard ratio (95% CI)	*p-*value	Hazard ratio (95%)	*p-*value
Age (≥70 years)	0.979 (0.596-1.607)	0.932		
Sex (male)	1.293 (0.760-2.200)	0.336		
Etiology (HCV)	1.288 (0.730-2.273)	0.373		
Child-Pugh (classification B or C)	0.866 (0.347-2.167)	0.755		
Cirrhosis	2.057 (0.978-4.323)	0.038	1.554 (0.714-3.382)	0.266
AFP (≥20 ng/mL)	1.683 (0.995-2.846)	0.060	1.223 (0.699-2.142)	0.480
DCP (≥40 mAU/mL)	1.174 (0.702-1.961)	0.544		
Ang2 (≥3.5 ng/mL)	1.680 (1.018-2.772)	0.045	1.633 (1.032-2.754)	0.041
Tumor numbers (multiple)	2.259 (1.331-3.834)	0.004	1.820 (1.049-3.155)	0.033
Maximal tumor diameter (>20 mm)	1.489 (0.900-2.466)	0.128		

## Abbreviations

AFP: alpha-fetoprotein
AUC: area under the curve
CLD: chronic liver disease
DCP: des-g-carboxy prothrombin
ELISA: enzyme-linked immunosorbent assay
HCC: hepatocellular carcinoma
OS: overall survival
RFA: radiofrequency ablation
RFS: recurrence-free survival
ROC: receiver-operating characteristics
SD: standard deviation
UICC: Union for International Cancer Control
